# The role of sub-ventricular zone in gliomagenesis

**DOI:** 10.18632/aging.100823

**Published:** 2015-10-19

**Authors:** Sara G.M. Piccirillo, Andrea Sottoriva, Colin Watts

**Affiliations:** University of Cambridge, Clinical Neurosciences, Cambridge, United Kingdom

**Keywords:** glioblastoma, sub-ventricular zone, cancer stem-like cells, intra-tumor heterogeneity, therapy resistance

Malignant gliomas, in particular glioblastoma (GBM), are incurable diseases characterised by high inter- and intra-tumor heterogeneity (ITH), diffuse brain infiltration and treatment resistance.

Over the last fifteen years, studies at the interface between stem cell biology and cancer research have established the existence of cancer stem-like populations in GBM and other brain tumors (reviewed in [1]). These data have been accompanied by speculation about neural stem cells being the cell of origin of these malignancies. In support of this hypothesis, mouse models studies have repeatedly suggested that GBM can originate from transformed neural stem/precursor cells [2-4]. However, similar evidence in human was still missing.

Recently, we have developed a real-time fluorescence-guided multiple sampling (FGMS) strategy to investigate the extent of spatial ITH in human GBM [5]. This approach stems from an initial proof-of-concept study aimed at assessing the use of a fluorescent marker (5-Aminolevulinic acid, 5-ALA) to investigate the distribution of cancer stem-like cells (alternatively named tumor propagating cells) at the core and margin of human GBM [6]. In our recent publication [7] we have used FGMS to identify visible fluorescence in the adult sub-ventricular zone (SVZ), a germinal region of the human brain and, for the first time, we have started to reveal its role in gliomagenesis and treatment resistance in humans.

Fluorescent disease was present in the SVZ of 65% of the GBM patients (in [7] we refer to this region as sub-ependymal zone because we could distinguish the ependymal layer separating the tissue from the ventricle by using an intra-operative fluorescent microscope). It is well established that 5-ALA fluorescence is specific to tumor cells in GBM. Therefore we hypothesised that visible fluorescence in the SVZ would identify malignant cells. The most common genetic alterations of GBM identified by The Cancer Genome Atlas (TCGA) were present in this region of all the analysed patients and genomic analysis showed ITH between the SVZ and matched tumor mass (T), thus confirming that the SVZ represents a niche of malignant cells. Interestingly, clustering of gene expression profiles revealed a marked predominance of mesenchymal signatures in almost 80% of the SVZ samples irrespective of the subtype of the corresponding T suggesting that the SVZ might nurture tumor growth.

To address the functional role of SVZ tumor cell populations, we characterised matched T- and SVZ-tumor propagating cells i*n vitro* and *in vivo*. We tested three drugs commonly used in GBM treatment: temozolomide (an alkylating agent that represents the standard of care for high grade glioma patients), cisplatin (an antimitotic agent) and cediranib (an antiangiogenic drug). Our analysis revealed three distinct generic patterns of drug response: a) differential response between T- and SVZ-cells, b) both cell populations responding to the drugs or c) neither of the two showing a good treatment response. Exposure to supra-maximal doses in chemo-naive patients was ineffective, reflecting the failure of dose-dense clinical studies and suggesting pre-existing resistant cell populations. These data shows how ITH impacts directly on treatment response and the emergence of resistant disease and confirms that the SVZ contains drug-resistant cells. These results have significant implications for treatment design and development of new therapeutic strategies.

**Figure 1:**
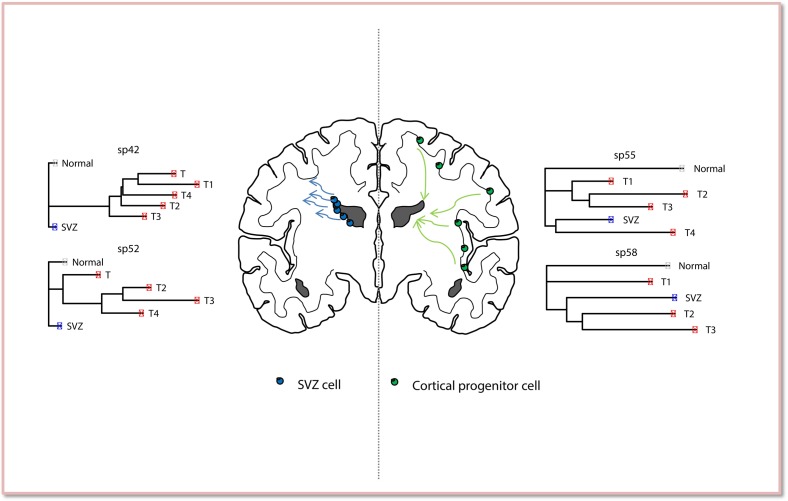
Phylogenetic analysis of human GBM reveals the role of the SVZ in gliomagenesis Two different evolutionary trajectories are observed supporting the hypothesis that GBM might originate either from neural stem/progenitor cells residing in the SVZ or from cortical progenitor cells. Representative examples of these two different scenarios are presented here (samples sp42, sp52, sp55, sp58).

Therapeutic targeting of the SVZ will require a more detailed understanding of its role in gliomagenesis. We have previously shown that phylogenetic analysis using FGMS permits the discrimination of early genetic alterations present at all sites of GBM versus later alterations that are present in only part of it [5]. This allows the resolution of the temporal ITH that occurs during GBM development. Phylogenetic analysis of 8 GBM patients revealed that the SVZ contains early clones in 4 out of 8 cases suggesting that this niche represents the “area of origin” of the tumor and also pointing to the neural stem/progenitor cells as the best candidate of malignant transformation. This is consistent with data from animal models [2-4]. However, a more complex scenario of disease evolution emerged in the remaining patients with the SVZ being characterised by late emerging clones, possibly originating from the malignant transformation of cortical progenitor cells (Fig. 1).

Our data reveals a complicated dynamic process of GBM evolution that involves multiple, genetically diverse clonal and sub-clonal populations involving both the SVZ and the tumor mass [8]. Given the poor efficacy of the standard line of treatments for GBM patients and the unrecognised involvement of the SVZ in gliomagenesis, we have focused our study on the patterns of response to treatments of SVZ- and Tcancer stem-like cells. The relevance of these findings lies in the unprecedented opportunity to improve our understanding of the impact of ITH on therapy response and GBM evolution and ultimately to the development of new therapeutic approaches targeting the SVZ and improving patients survival.
